# GM-CSF Quantity Has a Selective Effect on Granulocytic vs. Monocytic Myeloid Development and Function

**DOI:** 10.3389/fimmu.2018.01922

**Published:** 2018-08-28

**Authors:** Li Sun, Jai Rautela, Rebecca B. Delconte, Fernando Souza-Fonseca-Guimaraes, Emma M. Carrington, Robyn L. Schenk, Marco J. Herold, Nicholas D. Huntington, Andrew M. Lew, Yuekang Xu, Yifan Zhan

**Affiliations:** ^1^Anhui Provincial Key Laboratory for Conservation and Exploitation of Biological Resources, School of Life Science, Anhui Normal University, Wuhu, China; ^2^The Walter & Eliza Hall Institute of Medical Research, Parkville, VIC, Australia; ^3^Department of Medical Biology, University of Melbourne, Parkville, VIC, Australia; ^4^Department of Immunology and Microbiology, University of Melbourne, Parkville, VIC, Australia; ^5^Guangzhou Women and Children's Medical Centre, Guangzhou Institute of Pediatrics, Guangzhou Medical University, Guangzhou, China

**Keywords:** GM-CSF, dendritic cells, inflammation mediators, granulocytes, cytokines

## Abstract

GM-CSF promotes myeloid differentiation of cultured bone marrow cells into cells of the granulocytic and monocytic lineage; the latter can further differentiate into monocytes/macrophages and dendritic cells. How GM-CSF selects for these different myeloid fates is unresolved. GM-CSF levels can change either iatrogenically (e.g., augmenting leukopoiesis after radiotherapy) or naturally (e.g., during infection or inflammation) resulting in different immunological outcomes. Therefore, we asked whether the dose of GM-CSF may regulate the development of three types of myeloid cells. Here, we showed that GM-CSF acted as a molecular rheostat where the quantity determined which cell type was favored; moreover, the cellular process by which this was achieved was different for each cell type. Thus, low quantities of GM-CSF promoted the granulocytic lineage, mainly through survival. High quantities promoted the monocytic lineage, mainly through proliferation, whereas moderate quantities promoted moDCs, mainly through differentiation. Finally, we demonstrated that monocytes/macrophages generated with different doses of GM-CSF differed in function. We contend that this selective effect of GM-CSF dose on myeloid differentiation and function should be taken into consideration during pathophysiological states that may alter GM-CSF levels and during GM-CSF agonistic or antagonistic therapy.

## Introduction

Granulocyte-macrophage colony stimulating factor (GM-CSF) was so named, as it stimulates the production of granulocytes and macrophages from bone marrow (BM) cells (1). BM cells cultured in GM-CSF have also been used to produce dendritic cells *in vitro* (2, 3); such cells resemble monocyte-derived dendritic cells (moDCs) *in vivo* (4–6). Thus, GM-CSF could stimulate BM cells to differentiate into three myeloid subsets: granulocytes, monocytes/macrophages (mo/mΦ) and moDCs. The latter two populations are both monocytic myeloid cells, but mo/mΦ and moDCs derived from mouse BM cultured under GM-CSF belong as distinct entities (5). Even though there are differences between the classically circulating monocytes and tissue macrophages (7, 8), for the purpose of our study we have grouped cells derived from BM as monocytic myeloid cells and gated in flow cytometry as Ly6G^lo^CD11b^hi^, which can be further divided into mo/mΦ and moDCs phenotypically and functionally (e.g., increased expression of MHC-II, increased motility and more potent stimulation of CD4^+^ and CD8^+^ T cells) (5). How GM-CSF can differentially generate each of the three myeloid types has not been fully elucidated.

GM-CSF is not essential for normal haematopoiesis but is essential for maintenance of pulmonary surfactant homeostasis and emergency haematopoiesis that provide increased demand for granulocytes and macrophages to fight infection (9–11). Although GM-CSF is a potent cytokine driving *in vitro* differentiation of moDCs, it is thought to be not essential for *in vivo* moDCs differentiation (12, 13). Nevertheless, moDCs were significantly elevated in GM-CSF transgenic (GMtg) mice (14). The varied dependence of multiple myeloid cells on GM-CSF in different settings may reflect the levels of GM-CSF presented. Notably, during the infection with bacteria and parasite, the levels of GM-CSF are significantly elevated (15, 16). Similarly, the levels of GM-CSF were found to be significantly elevated in the serum and tissue of inflammatory diseases such as rheumatoid arthritis and colitis (17–19). Thus, GM-CSF levels change during infection and inflammation. Clinically, GM-CSF has been administered to accelerate leukopoietic recovery after myelosuppression from radio- or chemo-therapy or to mobilize leukopoietic cells into the circulation so that blood can replace BM as a source of precursor cells (20, 21). GM-CSF has also been advocated as an immune stimulant in cancer therapy. In this regard, one review concluded that immune stimulation occurred with low GM-CSF doses but often the opposite with high doses (22). GM-CSF antagonism (e.g., via anti-GM-CSF or GM-CSFR antibodies) are also undergoing clinical trials for treating inflammatory or autoimmune diseases (e.g., rheumatoid arthritis) (23, 24). Despite the pathophysiological and iatrogenic importance of GM-CSF, what effects of different levels of GM-CSF on various myeloid lineages remain undefined.

Here we dissected the effects of different doses of GM-CSF on the development of the three major myeloid cell types: granulocytes, mo/mΦ and moDCs. We investigated their cellular kinetics of survival, proliferation and differentiation. We also asked how different GM-CSF doses might alter the functional outcome. Our findings provide further insight into roles (sometimes paradoxical) ascribed to GM-CSF.

## Materials and methods

### Mice

C57BL/6 (B6, WT), CCR2.CFP.DTR, GM-CSF transgenic (GMtg) mice, and CCR2.CFP.DTR/GMtg (14, 25), A1^−/−^ mice (26), and Fucci (Fluorescence Ubiquitin Cell Cycle Indicator) mice (27) were housed under specific pathogen-free conditions at The Walter & Eliza Hall Institute of Medical Research. All experiments were performed in accordance with relevant guidelines and regulations that were approved by the Walter & Eliza Hall Institute of Medical Research animal ethics committee (Project #2014.023, #2016.014, #2017.008).

### Cell preparation, antibodies, and flow cytometry

Cells from spleen and pooled subcutaneous lymph nodes (inguinal, axial, brachial, cervical) unless specified were prepared by digestion in collagenase/DNase I as described (28). Single cell suspension was also prepared from lung and liver in some experiments. Antibodies (Abs) used in this study were CD4 (RM4-5, PE-Cy7, BV500), CD8 (53–6.7, Percp), anti-CD11c (HL3, APC, APC-Cy7), CD11b (M1/70, BV421, PE-Cy7), CD16/32 (2.4G2, APC-Cy7), CD24 (M1/69, PE), CD40 (3/23, PE), CD80 (10A1, PE), CD86 (GL1, PE), CCR2 (475301, APC, R&D systems), CD64 (X54-5/7.1, PE, APC), CD135 (A2F10, PE, eBioscience), CD115 (AFS98, APC, eBioscience), CD117 (2B8, APC), CD206 (C068C2, APC, Biolegend), CD326 (G8.8, AlexaFluor647, Biolegend, CCR7 (4B12, PE, eBioscience), CX3CR1 (SA011F11, APC, Biolegend), F4/80 (17-4801, eBioscience), FcεR1 (1-Mar, PE-Cy7), GM-CSF receptor α (698423, APC, R&D systems), I-A/I-E (M5/114.15.2, FITC, PE-Cy7), Ly6G (IA8 FITC, PE, PE-Cy7), Ly6C (AL-21, APC-Cy7, FITC, PE-Cy7), NK1.1 (PK136, PE), PD-L1 (M1H5, PE), and Rat IgG2b (PE, APC). All Abs were purchased from BD Biosciences except where stated otherwise, with cell numbers determined by the addition of fluorochrome-conjugated calibration beads (BD Biosciences, San Jose, CA) directly to the samples. For evaluation of expression level, fluorescene minus one (FMO) control was included. Data were collected using FACS Verse (BD Biosciences) and analyzed using FlowJo software (Tree Star, Ashland, OR). Cell sorting was performed by using a FACS Aria or an Influx cell sorter (BD Biosciences).

### BM cell culture

BM cells from mice were isolated by flushing femurs and tibias with 5 ml PBS supplemented with 2% heat-inactivated fetal bovine serum (FBS) (Sigma Aldrich, Lenexa, KS, USA). The BM cells were centrifuged once and then re-suspended in tris-ammonium chloride at 37°C for 30 s to lyse red blood cell. The cells were centrifuged again and then strained through a 70-μm filter before being re-suspended in RPMI-1640 supplemented with 10% FBS. For GM-CSF stimulated culture, BM cells were re-suspended at 0.5 × 10^6^/ml containing titrated doses of GM-CSF. After 3–4 days, the cultures were added fresh media with cytokines. Cell cultures were harvested on different day over 7 days.

### Cell survival assays

moDCs were enriched from spleen cells by using a Nycodenz density gradient as previously described (28) and further purified by flow sorting; granulocytes and monocytes from blood cells as *in vivo* source and from 7-day BM cultures with GM-CSF as *in vitro* source were purified by flow sorting. Purified cells were cultured at 1–5 × 10^4^ in 200 μL RPMI-1640 supplemented with 10% FBS in U-bottom 96-well plates in the absence or the presence of graded doses of GM-CSF. Upon harvesting, cells were stained for cell surface markers. Cell survival was measured by flow cytometry with FITC or APC-conjugated FACS calibration beads (BD Biosciences) and PI to determine the number of viable cells.

### Cell proliferation assays

BM or Blood cells from Fucci mice were cultured with or without CTV (Invitrogen, ThermoFisher, Waltham, MA) labeling (as per manufacturer's protocol). Labeled cells were cultured at 1 × 10^5^ in 200 μL RPMI1640 supplemented with 10% FBS in flat-bottom 96-well plates in the absence or the presence of graded doses of GM-CSF. Cell cultures were harvested every day and cells were stained for cell surface markers.

### Cell stimulation and cytokine assay

Myeloid cells (granulocytes, mo/mΦ, and moDCs)-derived from 7-day BM cultures were purified by flow sorting. Then cells were cultured at 5 × 10^4^ in 200 μL RPMI1640 supplemented with 10% FBS in U-bottom 96-well plates in the absence or the presence of LPS (1 μg/mL) or CpG (1 μM) for 20 h. For cytokine detection from the supernatants of *in vitro* assays, the indicated cytokines were detected using Cytometric Bead Array (CBA) technology (BD Biosciences) according to the manufacturer's instructions using a FACS Verse (BD Biosciences) cytometer.

### Statistical analysis

Statistical comparisons of mean difference between two groups from independent experiments were made using a student's *t*-test, and data presented as dose response curves or time courses were made using ANOVA. The analysis was performed with Prism v.5.0 software (GraphPad, San Diego, CA). *P* < 0.05 were considered statistically significant.

## Results

### High dose GM-CSF favors generation of monocytic myeloid cells while low dose GM-CSF sustains generation of granulocytes

**Figure 1:**
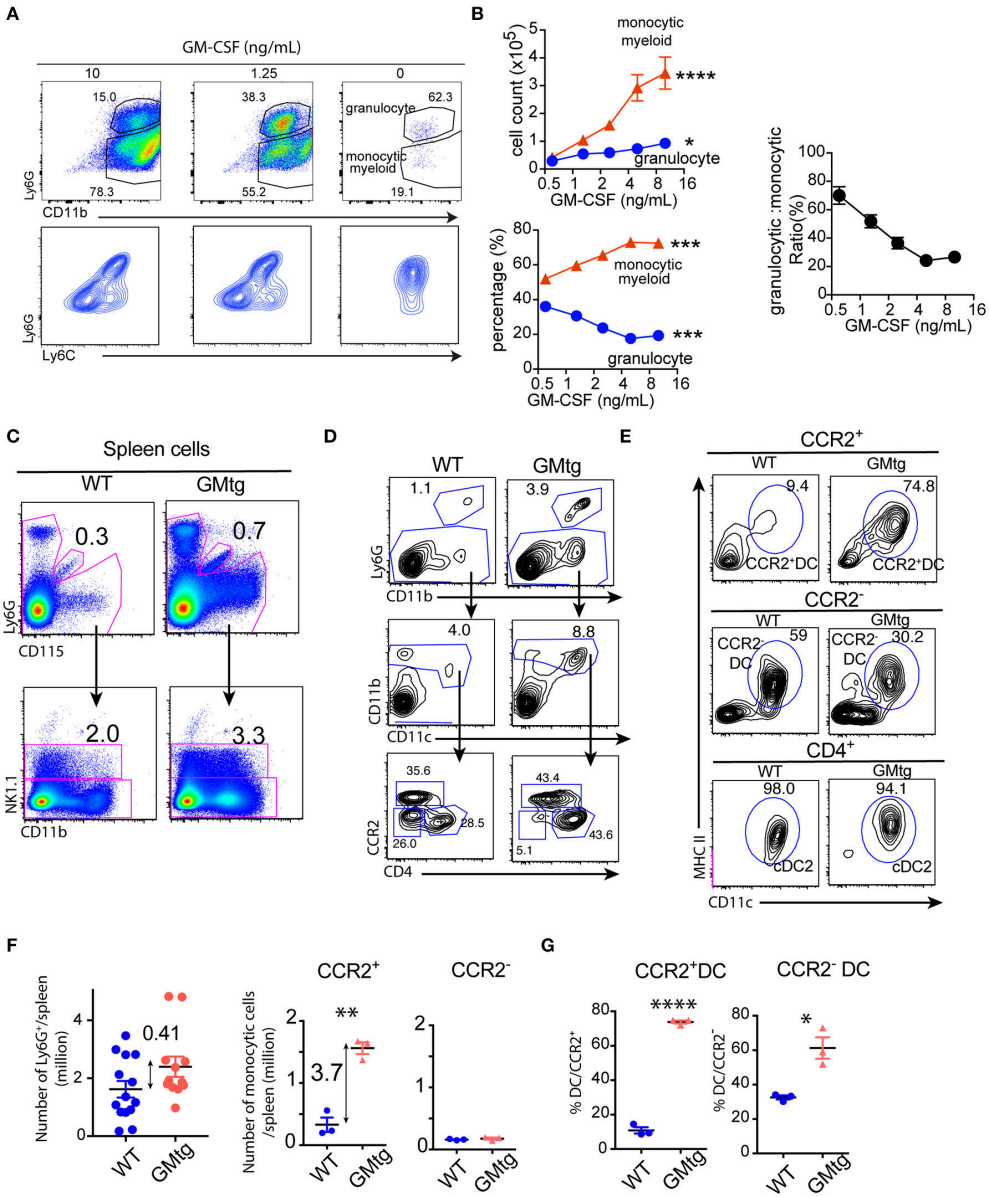
The effect of GM-CSF dose on generation of granulocytic vs. monocytic myeloid cells. BM cells were cultured in the presence of different doses of GM-CSF for 7 days. Cultures were harvested and analyzed for cell composition. **(A)** FACS plots show profiles of Ly6G^hi^ granulocytes and Ly6G^lo^CD11b^hi^ monocytic myeloid cells on gated PI^−^CD11b^+^ cells. **(B)** Plots show the recovered cell number and percentage of granulocytes and monocytic myeloid cells with different doses of GM-CSF. ^*^p < 0.05, ^***^p < 0.001, ^****^p < 0.0001 (ANOVA). Data are from one of 3 repeated experiments. Plot on the right shows the ratio of granulocyte to monocytic myeloid cells in the cultures with different doses of GM-CSF. **(C–G)** Spleen cells of WT (including CCR2.CFP.DTR mice) and GMtg mice (including CCR2.CFP.DTR/GMTg mice) were prepared and stained for myeloid markers. **(C)** autofluorescent macrophages and NK1.1^+^ cells were gated out for analysis of CD11b^+^ cells; **(D)** within CD11b^+^ cells, granulocytes were gated as Ly6G^+^ cells; Ly6G^−^ cells were then separated into three populations: CD11b^+^ CD4^+^ cDC2, CCR2^+^ and CCR2^−^ cells; **(E)** Three populations are shown for expression of CD11c and MHC class II. **(F)** Scatter plots show the number of granulocytes (pooled from 4 independent experiments) and monocytic myeloid cells (from one of 4 similar experiment). Numbers in the plots show fold increase. ^**^p < 0.01 **(G)** CCR2^+^ and CCR2^−^ cells are shown for the percentages of DCs. ^*^p < 0.05, ^****^p < 0.0001 (student's *t*-test).

To determine the effect of GM-CSF dose on the generation of granulocytic vs. monocytic myeloid cells, BM cells were cultured with different doses of GM-CSF (ranging from 0 to 10 ng/mL). Granulocytes were identified as Ly6G^hi^CD11b^+^, whereas monocytic myeloid cells as Ly6G^lo^CD11b^+^ (Figure 1A). When cultures were harvested after 7 days, the numbers of monocytic myeloid cells was up to 10-fold higher with higher doses of GM-CSF (Figure 1B). On the other hand, we observed that the numbers of granulocytes increased very modestly with increased doses of GM-CSF (Figure 1B). Proportionally, monocytic myeloid cells increased as dose of GM-CSF increased while granulocytes decreased as dose of GM-CSF increased (Figures 1A,B). Thus, the ratio of granulocytes to monocytic myeloid cells is inversely correlated to GM-CSF doses (Figure 1B).

To determine whether a similar trend occurred *in vivo*, we compared GM-CSF overexpressing mice (GMtg) with WT mice (with lower levels of endogenous GM-CSF). Previous studies showed that GM-CSF overexpression resulted in an increase in both Ly6G^+^ granulocytes and monocytic myeloid cells (14, 29). Nevertheless, we observed that increase in monocytic myeloid cells in GMtg mice was greater than increase in granulocytes in spleen (Figures 1C–G). To assess accurately the influence of GM-CSF overexpression on monocytic myeloid cells, spleen cells were analyzed after gating out autofluorescent macrophages and CD11b^+^NK cells (Figure 1C). Even after gating out CD4^+^CD11b^+^cDC2, there was a significant increase in CD11b^+^CCR2^+^ cells in spleen of GMtg mice while there was no clear increase in CD11b^+^CCR2^−^ cells (Figures 1D–G). Of note, majority (about 70%) of spleen CD11b^+^CCR2^+^ cells of GMtg mice was DC-like and positive for CD11c and MHC class II while CD11b^+^CCR2^+^ cells of WT mice contained only a small fraction (< 10%) of moDCs (Figure 1G). As expected, all CD11b^+^CD4^+^ cDC2 cells were positive for CD11c and MHC class II. Apart from spleen, there was a greater increase in monocytic myeloid cells in blood (Supplementary Figure 1B), lung (Supplementary Figure 1B) and liver (Supplementary Figure 1C). As Ly6C can be downregulated on monocytes/macrophages under high levels of GM-CSF (14, 29), expression of CCR2 reporter we also used to aid identification of monocytic myeloid cell subsets. When monocytic cells were further separated into CCR2^+^ (either Ly6C^hi^ and Ly6C^lo^) and Ly6C^−^CCR2^−^ cells, increase in CCR2^+^ cells in GMtg mice was more prominent (Supplementary Figures 1A–C). Together, both *in vitro* and *in vivo* data indicated that higher concentration of GM-CSF predominately increases monocytic myeloid cells.

### Medium dose of GM-CSF favors generation of moDCs

**Figure 2:**
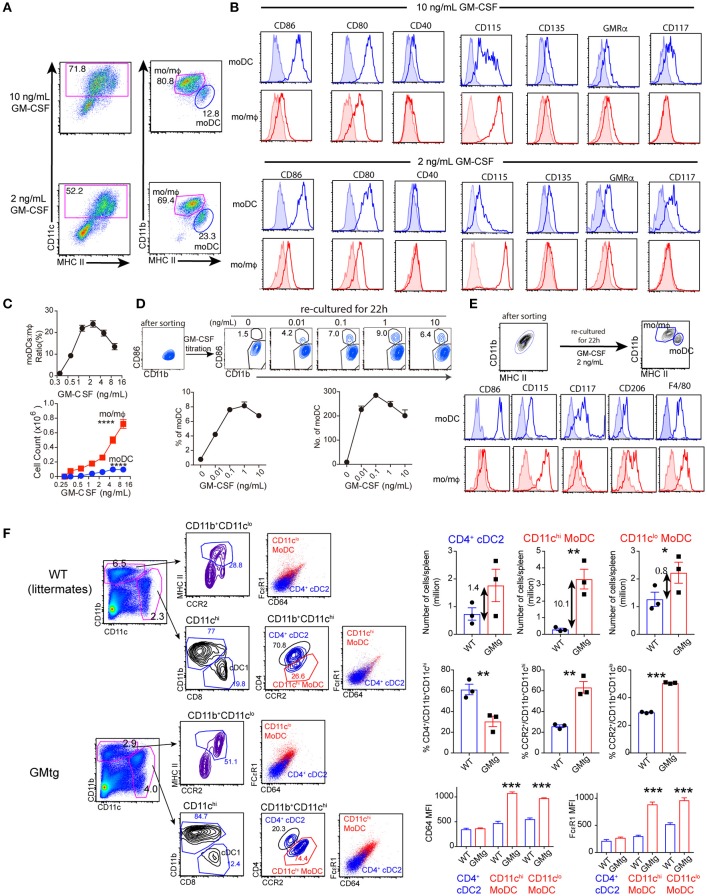
The effect of GM-CSF dose on the differentiation of mo/mΦ and moDCs. **(A–C)** BM cells were cultured with different dose of GM-CSF for 7 days. Cultures were harvested and analyzed for cell composition. **(A)**. Gating strategy for mo/mΦ and moDCs characterization in current study. **(B)** moDCs and mo/mΦ from gated Ly6G^lo^CD11c^+^MHCII^+^ population are shown for expression of additional markers. Shade area show FMO controls for gated population**. (C)** The ratio of moDCs to mo/mΦ and absolute number of recovered moDCs and mo/mΦ in the cultures with different doses of GM-CSF were plotted. ^****^p < 0.0001 (ANOVA). Data are from one of 3 repeated experiments. **(D)** After culturing with 10 ng/mL GM-CSF for 7 days *in vitro*, mo/mΦ were purified by flow sorting and then re-cultured with different doses of GM-CSF for 22 h. The percentage and number of recovered DCs are shown. **(E)** After culturing with 10 ng/mL GM-CSF for 7 days *in vitro*, sorted mo/mΦ were re-cultured with 2 ng/mL GM-CSF for 22 h. Histograms show expression of different markers of monocytic myeloid cells. Shade area show FMO controls for gated population. **(F)**. Spleen cells were prepared from CCR2.CFP.DTR mice (8–12 week female, *n* = 3) and CCR2.CFP.DTR/GMTg mice (8–12 week female, *n* = 3). FACS plots show distribution of moDCs and cDCs. Bar graphs show numbers, percentages and expression of CD64 and FcεR1 by DC subsets. Numbers in the plots show fold increase. ^*^p < 0.05, ^**^p < 0.01. *^**^p < 0.001 (student's *t*-test).

The monocytic myeloid lineage of BM cells under GM-CSF stimulation can further differentiate into mo/mΦ and moDCs, the latter being distinguished from the former by expression of higher levels of MHC class II but relatively lower expression of CD11b, defined as CD11c^+^MHCII^interm^CD11b^hi^ and CD11c^+^MHCII^hi^CD11b^interm^ respectively (5) (Figure 2A). As shown previously (5), we showed here that moDCs expressed higher levels of costimulatory molecules such as CD86, CD80 and CD40. They also expressed high levels of CD117, CD135, CD24 and CCR7, compared to mo/mΦ (Figure 2B and Supplementary Figure 2A). On the other hand, mo/mΦ expressed higher levels of CD115, F4/80, CD64, CD206, PD-L1, CCR2, and CXCR1 (Figure 2B and Supplementary Figure 2A).

Given our findings of granulocytic/monocytic deviation above, we decided to investigate whether GM-CSF dose may also determine mo/mΦ vs. moDCs fates within the monocytic myeloid cells. The highest proportion of moDCs occurred with intermediate doses of GM-CSF (1.25–5 ng/mL) (Figures 2A,C). The yield of mo/mΦ increased with increasing GM-CSF dose while yield of moDCs plateaued after 5 ng/mL (Figure 2C). Notably, moDCs developed under lower dose (2 ng/mL) and higher dose (10 ng/mL) expressed similar levels of DC markers CD86, CD80, CD135, and CD117. However, moDCs with lower doses expressed lower levels of some myeloid markers such as CD115, CD326 and CX3CR1, compared to moDCs generated with higher concentration of GM-CSF (Figure 2B and Supplementary Figure 2A). Furthermore, expression of GM-CSF receptor was higher on moDCs with lower GM-CSF (Figure 2B). Overall, differentiation of mo/mΦ and moDCs under GM-CSF stimulation show a subtle difference i.e., mo/mΦ increase with increasing GM-CSF dose but moDCs seem to be favored an intermediate dose.

As phenotypic analysis and transcriptome analysis concluded that mo/mΦ and moDCs derived from mouse BM cultured under GM-CSF belong as distinct entities (5), however, we observed some degree of plasticity for mo/mΦ converting to moDCs, at least based on expression of moDC markers. When mo/mΦ were isolated from 7-day BM cultures and further cultured with different doses of GM-CSF, we found that a substantial proportion of mo/mΦ can become moDCs (Figure 2D). Notably, higher proportion and number of DCs were observed with mid-range of GM-CSF concentration (Figure 2D). moDCs derived from these secondary cultures with GM-CSF showed similar expression of DC markers while remaining mo/mΦ maintained their identity (Figure 2E). We also observed such conversion when LPS, CpG or IL-4 was cultured with isolated mo/mΦ (data not shown). On the other hand, isolated moDCs maintained high expression of CD86 (data not shown).

Dissection of *in vivo* influence of GM-CSF on moDCs is complicated by two factors: that GM-CSF is not essential for development of moDCs (12, 25) and that moDCs share some myeloid markers of CD11b^+^ cDC2 and also downregulate certain markers such as Ly6C under higher GM-CSF and inflammation (30). Nevertheless, we compared spleen DC populations of WT and GMtg mice by using CCR2 reporter to aid identification of moDCs. In WT (CCR2.CFP.DTR) mice, the majority of spleen CD11c^hi^CD11b^+^ cells expressed CD4. However, CD11c^hi^CD11b^+^ cells also contained small fraction of CCR2^+^CD4^lo^ cells (likely moDCs, termed here as CD11c^hi^ moDCs). An additional moDC population expressing lower levels of CD11c also existed (termed as CD11c^lo^ moDCs) (Figure 2F). Although both population of moDCs expressed higher levels of CD64 and FcεR1, these two markers did not clearly separate them from CD4^+^ cDC2. In CCR2.CFP.DTR/GMtg mice, it was evident that CD11c^hi^CD11b^+^ cells contained an increased population of CCR2^+^CD4^lo^ cells. Similarly, CD11c^lo^ moDCs that express CCR2 also increased. Both populations also showed increased expression of CD64 and FcεR1 (Figure 2F). Together, elevated levels of GM-CSF also preferentially increased moDCs *in vivo*. Due to wide distribution of mo/mΦ in various tissues relative to DCs, it remains to be established whether elevated levels of GM-CSF differentially impact on DCs vs. mo/mΦ.

### GM-CSF dose has differential effects on survival of myeloid cells

**Figure 3:**
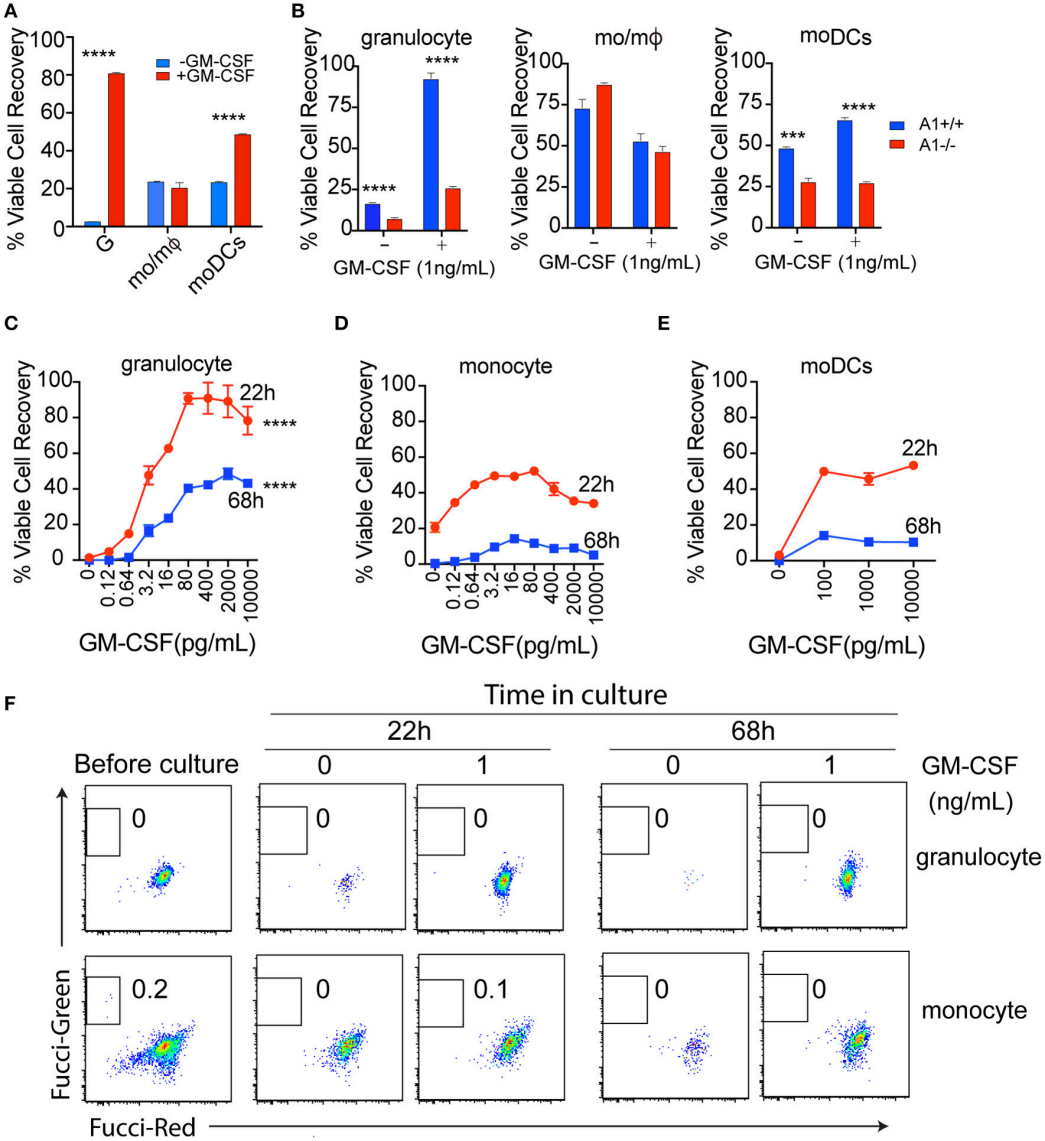
The effect of GM-CSF dose on myeloid cell survival in culture. **(A)** Granulocytes, moDCs and mo/mΦ were purified from WT BM cultures by flow sorting, then were cultured with or without 10 ng/mL GM-CSF for 22 h. The total recovery of viable (PI excluding) cells was presented. ^****^p < 0.0001 (student's *t*-test). Data are from one of 3 repeated experiments. **(B)** Granulocytes, moDCs and mo/mΦ were purified from WT and A1^−/−^ BM cultures by flow sorting, then were cultured with or 1 ng/mL GM-CSF for 22 h. The total recovery of viable (PI excluding) cells was presented. ^***^p < 0.001, ^****^p < 0.0001 (student's *t*-test). Data are from one of 3 repeated experiments. **(C,D)** Granulocytes and monocytes were isolated and purified from WT mice blood, then cultured with or without different doses of GM-CSF for 22 and 68 h. ^****^p < 0.0001(ANOVA). Data are from one of 3 repeated experiments. **(E)** moDCs were isolated and purified from GMtg mice spleen, then cultured with or without different doses of GM-CSF for 22 or 68 h. **(F)** To exclude the possibility that increased recovery of viable cells under GM-CSF stimulation was due to cell proliferation, granulocytes and monocytes from Fucci mice blood were isolated and purified, then cultured with or without different doses of GM-CSF. Data are from one of 3 repeated experiments.

One of the determinants of population size is cell survival. To examine the effect of GM-CSF on survival of generated granulocytes, mo/mΦ, and moDCs from BM cultures, we checked viability of the three myeloid cells in the presence or absence of GM-CSF at the end of culture period. We found that granulocytes had poor spontaneous survival in contrast to higher and similar survival rate of mo/mΦ and moDCs. In response to GM-CSF stimulation, however, the survival of both granulocytes and moDCs increased significantly, although the fold of increase were less in moDC than that of granulocytes. On the contrary, additional GM-CSF did not alter the survival of mo/mΦ (Figure 3A).

As A1 is an anti-apoptotic protein that can be induced by GM-CSF (30), we investigated the contribution of A1 to cell survival regulated by GM-CSF. To the end, we isolated granulocytes, mo/mΦ and moDCs generated from BM cultures of WT and A1^−/−^ mice. *In vitro*, survival of three types of cells under different doses of GM-CSF was evaluated. We found that A1 deficiency significantly reduced survival by granulocytes and moDCs with or without GM-CSF. On the other hand, survival of mo/mΦ was not grossly affected by A1 deficiency (Figure 3B).

To further confirm our *in vitro* findings, we isolated blood granulocytes and monocytes, then cultured them with different doses of GM-CSF. Without GM-CSF, granulocytes died rapidly in culture. Addition of as little as 3 pg/mL GM-CSF markedly increased granulocytes viability, reaching a plateau at 80 pg/mL over 1–3 days of culture (Figure 3C). Compared with granulocytes, monocytes had better spontaneous survival at least for the first 22 h of culture; addition of GM-CSF also increased monocytes survival (Figure 3D). However, GM-CSF did not sustain survival of monocytes. Majority of mo/mΦ died by 3 days even with GM-CSF. moDCs are scarce in normal mice, but are abundant in GMtg mice (14). Therefore, we isolated moDCs from the spleens of GMtg mice and cultured them with varying doses of GM-CSF. We found that 100 pg/mL GM-CSF had already achieved maximal levels of moDCs survival (Figure 3E). Notably, survival pattern was similar between monocytes and moDCs.

To exclude the possibility that increased recovery of viable cells under GM-CSF stimulation in the above blood samples was due to cell proliferation, we cultured blood leukocytes from mice with cell cycle reporters (Fucci mice, whereby green depicted cells in S, G2, and M phase whereas red depicted cells in G0 and G1 phase) with different doses of GM-CSF. Unsurprisingly, we did not find cells that were actively proliferating i.e., there was a lack of FucciGreen^+^ cells (Figure 3F). When moDCs from GMtg mice were evaluated for their proliferative potential, they did not proliferate with or without added GM-CSF (Supplementary Figure 1D), suggesting that increase in moDCs in GMtg mice is not due to expansion of terminally differentiated DCs.

### Dose of GM-CSF has differential effects on proliferation of monocytic myeloid cells and granulocytes

**Figure 4:**
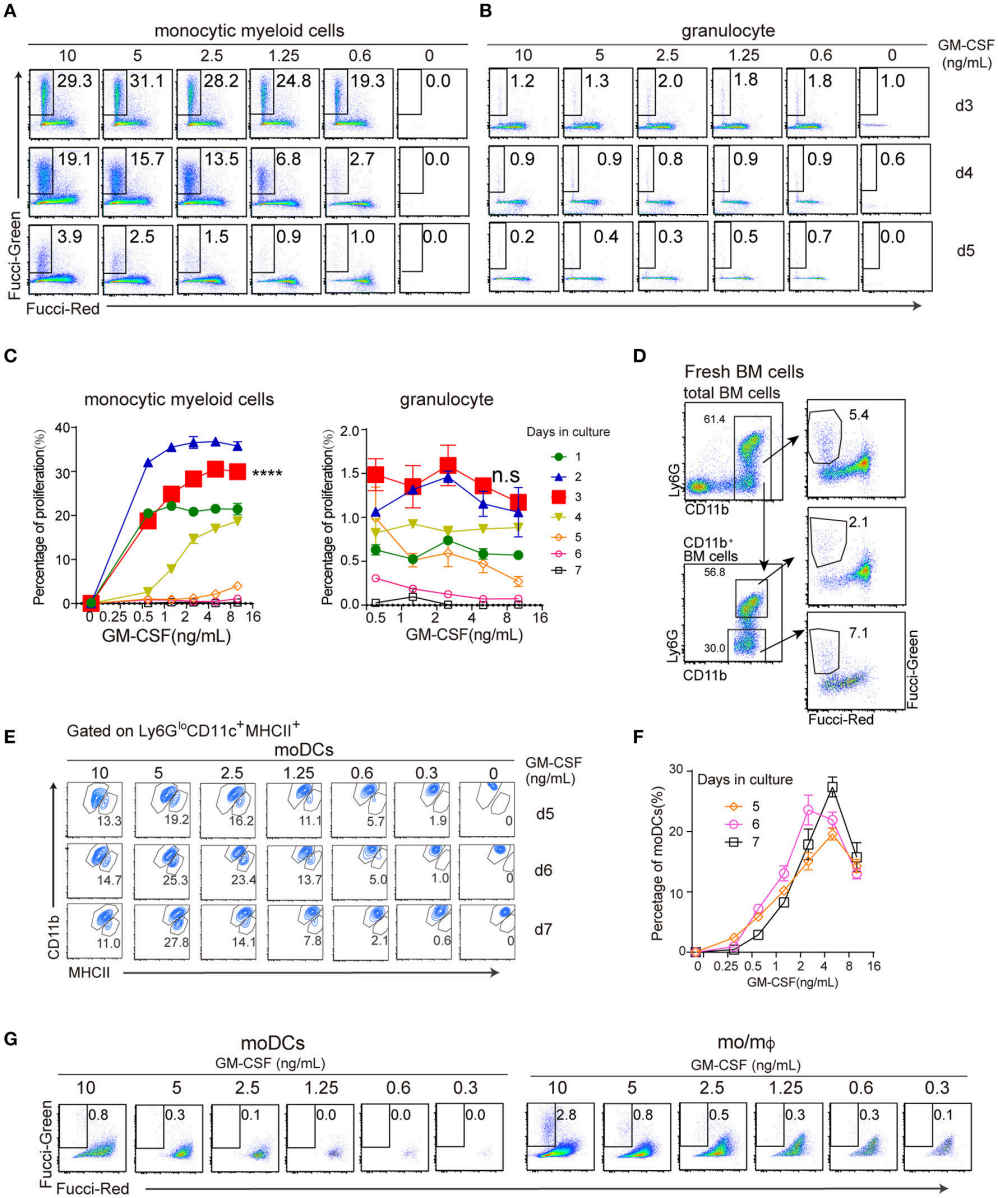
The effect of GM-CSF dose on the proliferation of monocytic myeloid cells and granulocytes. **(A,B)** FACS plots show profiles of FucciGreen^+^ Red^−^ proliferating cells of monocytic myeloid cells and granulocytes with different doses of GM-CSF from day3 to day5. **(C)** Plots show the percentage of proliferation of monocytic myeloid cells and granulocytes with different doses of GM-CSF from day1 to day7. ^****^p < 0.0001 (ANOVA). **(D)** Plots show BM cells prior to culture. **(E)** Gated on CD11c^+^MHCII^+^, FACS plots show profiles of MHCII^interm^CD11b^hi^ mo/mΦ and MHCII^hi^CD11b^interm^ moDCs with different doses of GM-CSF from day 5 to day 7. **(F)** Plots show the percentage of moDCs under different GM-CSF dose stimulation from day 5 to day 7. **(G)** FACS plots show profiles of proliferating cells of moDCs and mo/mΦ with different doses of GM-CSF on day5.

To track cell proliferation, we used BM cells from Fucci mice. Cultures of BM cells with different doses of GM-CSF were analyzed from 1 to 7 days (Figures 4A–C). For both cell types, cycling occurred from day 1, peaked around day 2–3 and ceased at day 5. There was a quantitative difference between monocytic myeloid cells (Ly6G^lo^CD11b^hi^) vs. granulocytes (Ly6G^hi^). We found that the proportion of cycling cells was much higher in monocytic myeloid cells than granulocytes over 7 days in culture; for example, on day 3 after culture with 5 ng/mL GM-CSF, 30% of monocytic myeloid cells were cycling, compared with 1.3% of granulocytes. Moreover, proliferation by monocytic myeloid cells (Figure 4C) increased with increased GM-CSF dose (*p* < 0.0001), whereas proliferation by granulocytes was similar regardless of GM-CSF dose at least for the range between 0.5 and 10 ng/mL (Figure 4C). Of note, fresh BM cells contained a cohort of Fucci-Green cells within CD11b^+^ fraction; about 7% of Ly6G^−^ and 2% Ly6G^+^ were positive for Fucci-Green (Figure 4D). Upon culture without growth factor, such populations disappeared (Figure 4A).

We note that appearance of moDCs within monocytic myeloid cells occurred later (observable after day 4). By then the rate of proliferation of monocytic myeloid cells had already reduced. Over 5–7 days in culture, the proportion of moDCs was highest when intermediated doses of GM-CSF (2.5–5 ng/mL) were used (Figures 4E,F). We also checked the viability of the moDCs and found that the survival of the moDCs was not affected by the doses of GM-CSF (Supplementary Figure 2B). Nevertheless, when moDCs and mo/mΦ were compared for proliferation rate at day 5, mo/mΦ contained more FucciGreen^+^Red^−^ cells than moDCs (2.8 vs. 0.8%; Figure 4G).

Overall, high doses of GM-CSF favored the proliferation of mo/mΦ. Most moDCs were differentiated after active proliferation stopped. Thus, appearance of moDCs between day 5 and 7 most likely represented differentiation. The rate of moDCs differentiation was highest with intermediate doses of GM-CSF.

### Granulocyte, mo/mϕ, and moDC from the same cultures differ in cytokine production

**Figure 5:**
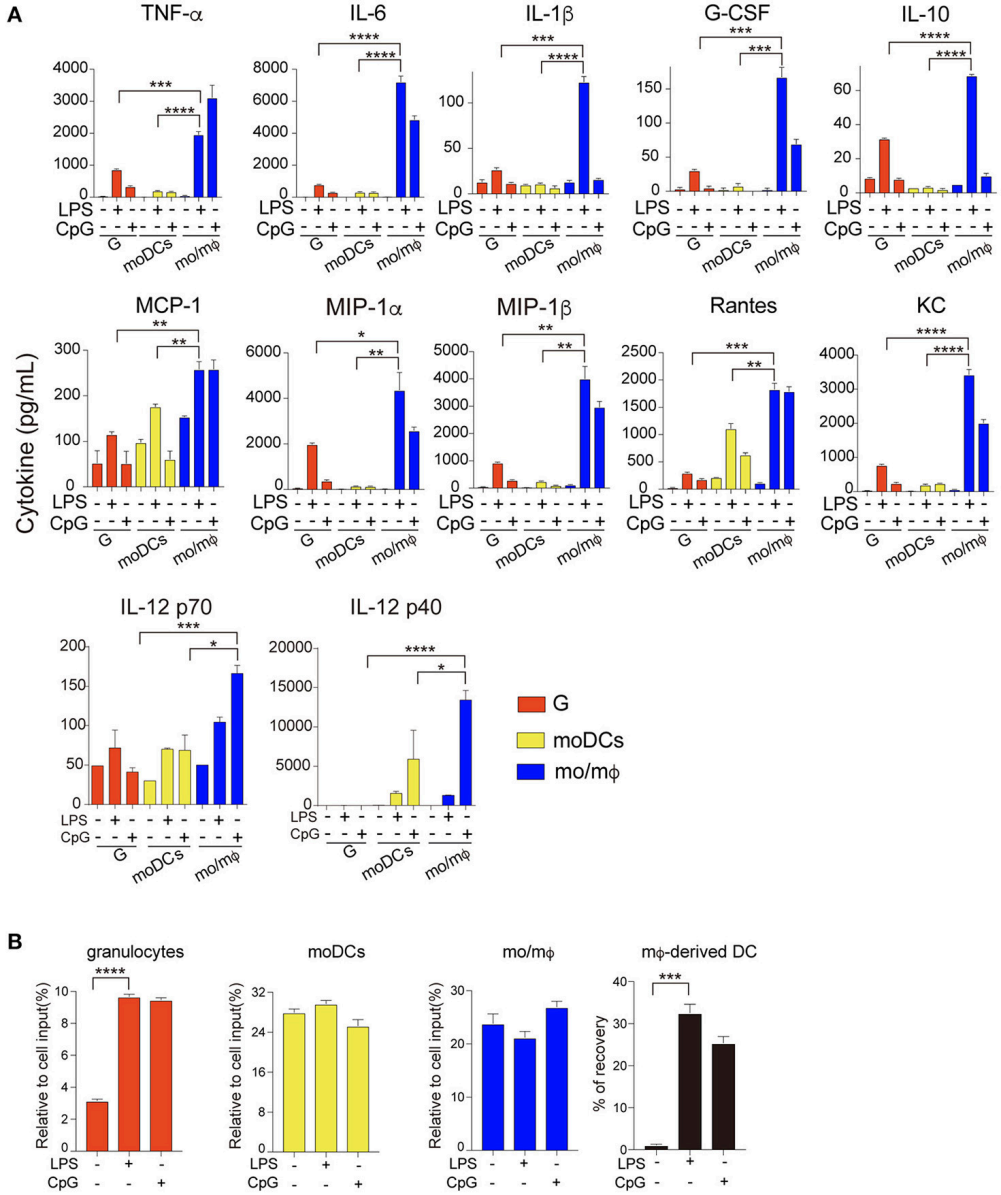
Granulocyte, moDCs, and mo/mΦ from the same cultures differ in cytokine production. After culturing with 10 ng/mL GM-CSF for 7 days *in vitro*, granulocytes (G), moDCs and mo/mΦ were purified by flow sorting, then stimulated by LPS (1 μg/mL) and CpG (1 μ) for 20 h. **(A)** Accumulation of inflammatory cytokines and chemokines in the supernatant was measured after 20 h. The concentration (pg/mL) of inflammatory cytokines and chemokines produced by the three myeloid populations are shown. **(B)** Plots show the percentage of cell recovery for the three populations after culturing for 20 h. ^*^p < 0.05, ^**^p < 0.01, ^***^p < 0.001, ^****^p < 0.0001(student's *t*-test).

In a previous report, cells generated in GM-CSF stimulated cultures may differ in function, including the production of certain cytokines by mo/mΦ and dendritic cells (5). Here we also included granulocytes. In accordance with that report, we found that among the three myeloid populations under LPS and CpG stimulation, mo/mΦ were the most potent producers of all 12 cytokines/chemokines measured (Figure 5A). Granulocytes were intermediate in their production of cytokines/chemokines (Figure 5A). The caveat here is that cell survival for the three populations varied: recovery of granulocytes was < 10% while recovery of moDCs and mo/mΦ was about 25% (Figure 5B). Of note, cultures of mo/mΦ under stimulation with CpG or LPS contained a fraction of moDCs. Together, taking differential survival (about 2-fold) and moDC contamination in consideration, mo/mΦ were still more potent producer of certain cytokines (for example IL-6).

Mo/mΦ can be generated with low or high dose of GM-CSF, albeit with different yield. When isolated mo/mΦ were stimulated with various doses of LPS (0, 1, 10 ng/mL) for 20 h, mo/mΦ generated with high dose GM-CSF in most cases produced higher levels of cytokines/chemokines than those generated with low dose GM-CSF (not shown). As survival of mo/mΦ can be impacted by LPS, we found out that survival or death of mo/mΦ generated with low or high dose of GM-CSF behaved similarly in response to TLR stimulation (not shown). However, we found that overall spontaneous survival of mo/mΦ generated with low GM-CSF was poor than those generated with high dose of GM-CSF (not shown), implying that reduced survival by mo/mΦ generated with low GM-CSF could contribute to low production of cytokines.

## Discussion

GM-CSF plays an important role in inflammation and immunity to pathogens, cancer and inflammation. It is released during emergency leukopoiesis and its main therapeutic use is to hasten leukopoiesis after BM suppression. Its influence in the development of multiple types of myeloid cells from BM cells has been extensively documented. Yet, how GM-CSF differentially affects each myeloid cell type has not been defined. Here we showed that GM-CSF quantity had a selective effect on which cell type was favored; moreover, the cellular process by which this was achieved was different. High doses of GM-CSF favor mo/mΦ production, largely through enhancement of cell proliferation while high doses of GM-CSF did not further increase proliferation of Ly6G^+^ cells that was induced with lower doses of GM-CSF. Granulocytes had very poor survival that were greatly improved with low concentration of GM-CSF while mo/mΦ had better spontaneous survival and addition of GM-CSF only moderately improved survival. We also revealed that differentiation of moDCs from monocytic cell population occurred at intermediate levels of GM-CSF. This differentiation occurred relatively late (from day 4 in the culture).

Our *in vitro* data indicate that GM-CSF quantity had a selective effect on differentiation of granulocyte and macrophages. High dose of GM-CSF mainly increases the production of monocytic myeloid cells. The finding is also largely vindicated *in vivo*. Two previous reports had showed that high doses GM-CSF lead to an increase in the monocytic:granulocytic ratio, even though they did not look at monocytic:granulocytic ratio directly. One study showed that their GMtg mice had >25-fold higher levels of circulating GM-CSF (31); such high levels would then explain the marked accumulation of monocytes in multiple organs (e.g., increase in granulocytes was much less conspicuous) (31). In another study where two lines of GMtg mice with different levels of GM-CSF were examined, it was the strain with higher GM-CSF that had the greater increase in spleen monocytes than granulocytes (32). In a recent study with GM-CSF overexpression, increase in monocytic myeloid cells in brain tissue was more prominent (29). Here we compared the two lineages of myeloid cells in WT (representing low levels of endogenous GM-CSF) and GMtg (representing elevated GM-CSF) mice. Although both types of cells were increased in GMtg mice, increase in monocytic myeloid cells (Ly6G^−^CD11b^+^), particularly CCR2^+^ cohort in blood, spleen, lung, and liver was larger in GMtg mice. We also observed previously that GM-CSF deficiency had differential effect on macrophages over neutrophils during listerial infection (10). Thus, elevated GM-CSF *in vivo* also differentially impact on two lineages of myeloid cells.

We further examined the impact of GM-CSF doses on proliferation of two lineages using mice with cell cycle reporters. In general, most cell proliferation occurred in the first 4 days of culture. Even with replenishment of fresh media, cell proliferation was not remarkably increased after 4 days. This probably reflects depletion of proliferating progenitors and limited proliferating capacity of fully differentiated myeloid cells in culture. During the first 4 days, proportion of cells actively cycling (FucciGreen^+^) in monocytic myeloid (Ly6G^lo^CD11b^high^) cells was positively correlated to GM-CSF doses. On the other hand, proportion of cells actively cycling (FucciGreen^+^) in Ly6G^hi^ cells reached a plateau with much lower doses. The proliferation data provides an explanation of how mo/mΦ generation is favored.

In addition, we investigated the impact of GM-CSF doses on survival of granulocytes and mo/mΦ. Granulocytes in culture rapidly lost viability and addition of GM-CSF greatly improved cell survival (33). Similar to what described for eosinophils (34), we found that only small quantities of GM-CSF (80 pg/mL) was required to achieve maximal survival enhancement for blood granulocytes. On the other hand, blood monocytes had better spontaneous survival in culture, but survival enhancement by GM-CSF was less remarkable, particularly at day 3 in culture. A similar trend was also observed when granulocytes and mo/mΦ were isolated from BM cultures with GM-CSF. At the molecular level, granulocytes rapidly lose the anti-apoptotic protein, MCL-1, in culture; GM-CSF could maintain MCL-1 stability and thus promote granulocyte survival (35). Although it was not a direct comparison, rapid loss of MCL-1 expression in cultured monocytes and monocytic cell line was found to be less evident (36). Given that GM-CSF induces the expression of anti-apoptotic protein A1 (37) and that A1 promotes granulocyte survival (38, 39), we investigated the contribution of A1 to survival of three types of myeloid cells. We found that *in vitro* survival of A1-deficient granulocytes and A1-deficient moDCs but not mo/mΦ was defective. Notably, recent characterization of mice lacking all functional isoforms of A1 revealed minor defect in granulocyte survival *in vitro* (40). Some of these discrepancies can be related to experimental conditions. Recent study on A1^−/−^ cells was performed on GR1^+^ bone marrow cells (40) while other two studies used blood/peritoneal granulocytes (38) or BM-derived granulocytes (39).

GM-CSF has been used for a long time as a cytokine critical for moDCs differentiation in BM cultures (2, 3, 5). We found that moDCs emerged rather late compared with mo/mΦ (after day 5). This perhaps is not surprising, as much cell proliferation has ceased by then, as indicated by the lack of FucciGreen^+^ cells. Thus, moDCs represent bona fide differentiation from Ly6G^lo^CD11b^hi^ monocytic myeloid cells. As discussed above, generation of CD11b^hi^ monocytic myeloid cells that contain both mo/mΦ and moDCs was directly correlated with GM-CSF dose. We noticed that moDCs proportion started to reduce when GM-CSF concentration was high (>10 ng/mL); an intermediate dose of GM-CSF favored moDCs differentiation. In support, there was a recent study showing that high concentration of GM-CSF (10–100 ng/mL) favored macrophage differentiation (41). Our data and data from the recent study (41) are somewhat different from a previous study(42) showing that DCs generated with low GM-CSF (5 U/mL) expressed low levels of CD86 and represent immature DCs while DCs generated with high (100 U/mL) GM-CSF expressed high levels of CD86. It is difficult to reconcile these contradicting findings, except likely contribution of variation in actual GM-CSF activity and culture condition. Nevertheless, it highlights that GM-CSF dose used to generate DCs has profound impact on abundance and functionality of generated DCs.

In our study, we isolated mo/mΦ and moDCs from GM-CSF stimulated cultures for 7 days and cultured further with GM-CSF and fresh media. In the secondary cultures, moDCs maintained their phenotype while proportion of mo/mΦ could turn into moDCs. This would suggest that moDCs were terminally differentiated whereas at least a substantial proportion of mo/mΦ might not be. It has been reported that *in vivo* isolated Flt3^+^CD11c^−^ MHCII^+^ PU.1^hi^ monocyte subset can act as precursors of GM-CSF dependent moDCs (43). It is currently unknown whether mo/mΦ fraction from GM-CSF stimulated BM cultures contains a defined population of precursors with DC potential. It is previously reported that mo/mΦ cells from *in vitro* GM-CSF stimulated BM cultures might also contain “DC-precursors” that expressed intermediate levels of MHC class II although there was no clear “subpopulation” based on expression of MHC class II in mo/mΦ population (44). These cells can further upregulate MHC class II in the presence of GM-CSF (44). Thus, it is likely that transition of mo/mΦ to moDCs was not soly a stochastic process.

GM-CSF dose likely has different impact on cell function in several ways. Firstly, GM-CSF dose generates different myeloid cells with different function. mo/mΦ and moDCs generated from the same cultures have been reported to greatly differ in cytokine production and T cell priming (5). We extended such comparisons by the inclusion of granulocytes and by titrating the dose of GM-CSF. Overall, mo/mΦ are the most potent producers of cytokines and chemokines. Interestingly, mo/mΦ generated with M-CSF were less potent than mo/mΦ generated with GM-CSF for production of most pro-inflammatory cytokines (45). Thus, mo/mΦ generated with GM-CSF resemble M1 mΦ while mo/mΦ generated with M-CSF with high IL-10 production resemble M2 mΦ (45). Secondly, different GM-CSF dose has differential effects on survival of three myeloid cells and therefore indirectly affect cell function. In this regard, we also found that mo/mΦ generated with low GM-CSF is less fit and survive poorer than mo/mΦ generated with high GM-CSF with or without TLR stimulation. Thirdly, GM-CSF can directly affect cell function. We showed here that mo/mΦ generated with high dose of GM-CSF were more potent in generating cytokines and chemokines. Fittingly, *in vivo* GM-CSF overexpression also resulted in highly activated mo/mΦ causing severe inflammation (31).

The precise mechanism affecting cell differentiation and function by GM-CSF is not well-understood. Given that GM-CSF signaling can lead to the activation of multiple intracellular signaling modules, including JAK/STAT, MAPK, PI3K, it may be that different GM-CSF dose has different impact on the balance of these signaling events. Transcriptome analysis also revealed that GM-CSF induces multiple cellular pathways required for function of inflammatory monocytes of mouse and human (46). How GM-CSF induced-transcriptome changes are differentially affected by doses of GM-CSF would be of interest to researcher in the field in order to understand the requirement of GM-CSF for development of pathogenic function. Recently, it has been reported that activation of the aryl hydrocarbon receptor (AHR) promoted moDC differentiation while impairing differentiation into mo/mΦ (47). It would also be interesting to test whether GM-CSF dose alter AHR activation.

Together, we demonstrated that GM-CSF dose acts as a rheostat regulating survival, proliferation, differentiation and function of three myeloid cell types generated under GM-CSF stimulation. These findings could provide some answers why different GM-CSF doses could be either immunostimulatory or immunosuppressive (48); why GM-CSF could be either promoting autoimmunity (49) or inhibiting autoimmunity (50). Given that GM-CSF levels can change greatly from steady state to inflammation or severe infection, our findings may be useful in guiding the understanding of such pathophysiological conditions as well as management during GM-CSF agonist or antagonist therapy.

## Author contributions

YZ, YX, AL, and LS designed research. LS, JR, RD, FS-F-G, and EC performed research. RS and MH contributed vital new reagents or analytical tools. LS, JR, RD, FS-F-G, EC, and RS collected, analyzed, and interpreted data. LS, YZ, YX, AL, and NH wrote the paper.

### Conflict of interest statement

The authors declare that the research was conducted in the absence of any commercial or financial relationships that could be construed as a potential conflict of interest.
