# Supplementary material for: GM-CSF Quantity Has a Selective Effect on Granulocytic vs. Monocytic Myeloid Development and Function
Source: Front Immunol. 2018 Aug 28;9:1922. doi: 10.3389/fimmu.2018.01922 (PMC6120981; doi:10.3389/fimmu.2018.01922)
Supplement: Supplementary file 1 [file Image_1.PDF]

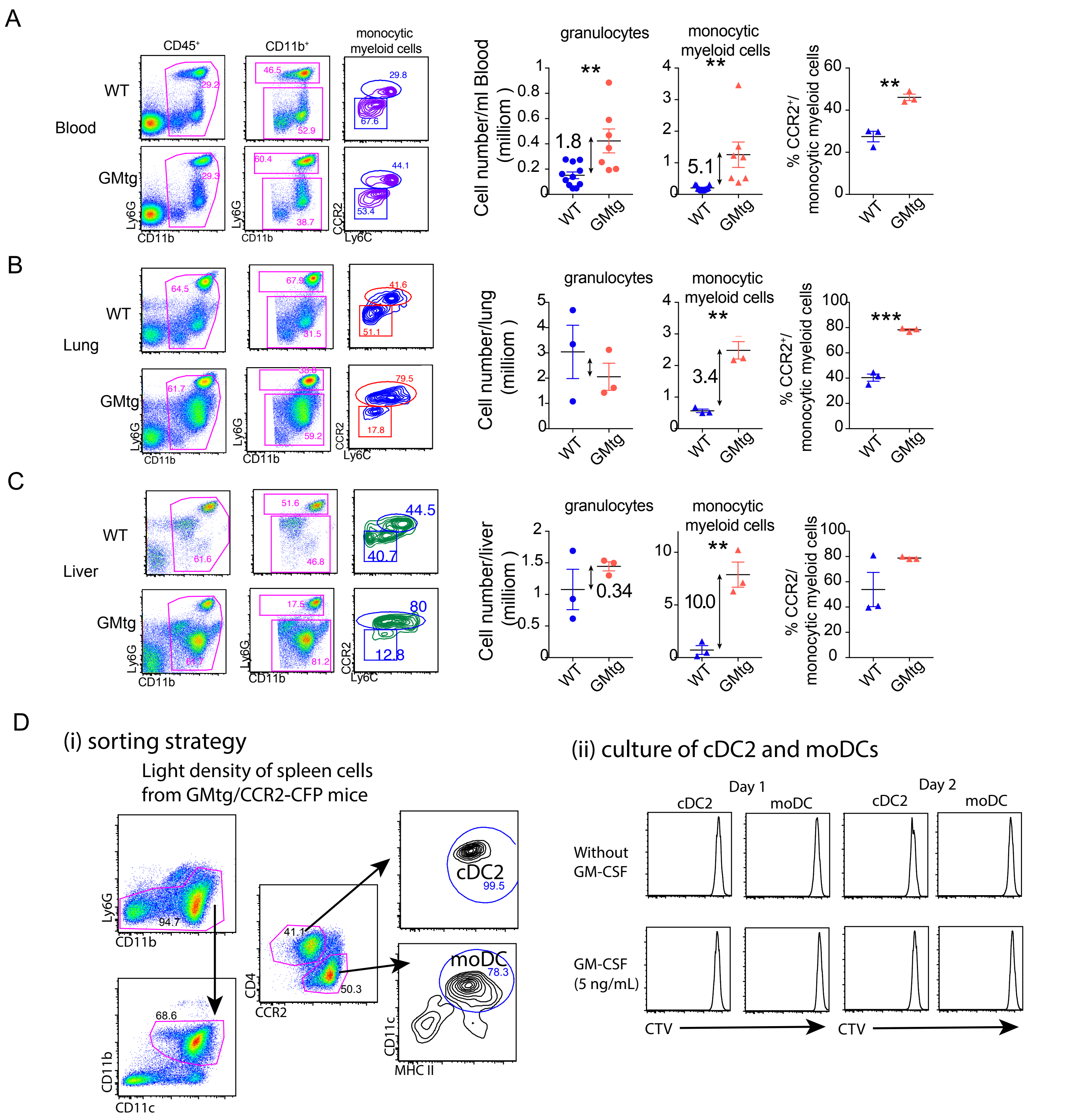

Supplemental Figure 1:

The effect of GM-CSF dose on generation of granulocytic versus monocytic myeloid cells.

A-C: Leukocytes from blood, lung and liver of WT (including CCR2.CFP.DTR mice) and GMtg mice (including CCR2.CFP.DTR/GMTg mice) were prepared and stained for myeloid markers. Blood leukocytes (A) were from WT (8-12 week female, n=11) and GMtg mice (8-12 week female, n=9); Lung (B) and liver (C) were from CCR2.CFP.DTR mice (8-12 week female, n=3) and CCR2.CFP.DTR/GMTg mice (8-12 week female, n=3). FACS plots show distribution of Ly6G^{+} and Ly6G^{-}CD11b^{+} populations.
Ly6G^{-}CD11b^{+} population was then shown for expression of CCR2 reporter and Ly6C. Scatter plots show the number of granulocytes and monocytic myeloid cells within gated CD11b^{+} populations and percentage of CCR2^{+}(Ly6C$^{hi/lo}$) out of total Ly6G^{-}CD11b^{+} population. Numbers in the plots show fold increase. *p<0.05, **p<0.01 (student's t test). Data are pooled from 3 independent experiments.

D. moDCs from GMtg mice do not proliferate with or without GM-CSF. Spleen cells were prepared from CCR2.CFP.DTR mice and CCR2.CFP.DTR/GMTg mice. Light density cells were sorted for cDC2s and moDCs (i). CTV labelled cells were then cultured with or without GM-CSF for 1-2 days. Histograms show CTV profile of cultured cells.
