# Supplementary material for: GM-CSF Quantity Has a Selective Effect on Granulocytic vs. Monocytic Myeloid Development and Function
Source: Front Immunol. 2018 Aug 28;9:1922. doi: 10.3389/fimmu.2018.01922 (PMC6120981; doi:10.3389/fimmu.2018.01922)
Supplement: Supplementary file 2 [file Image_2.PDF]

A.

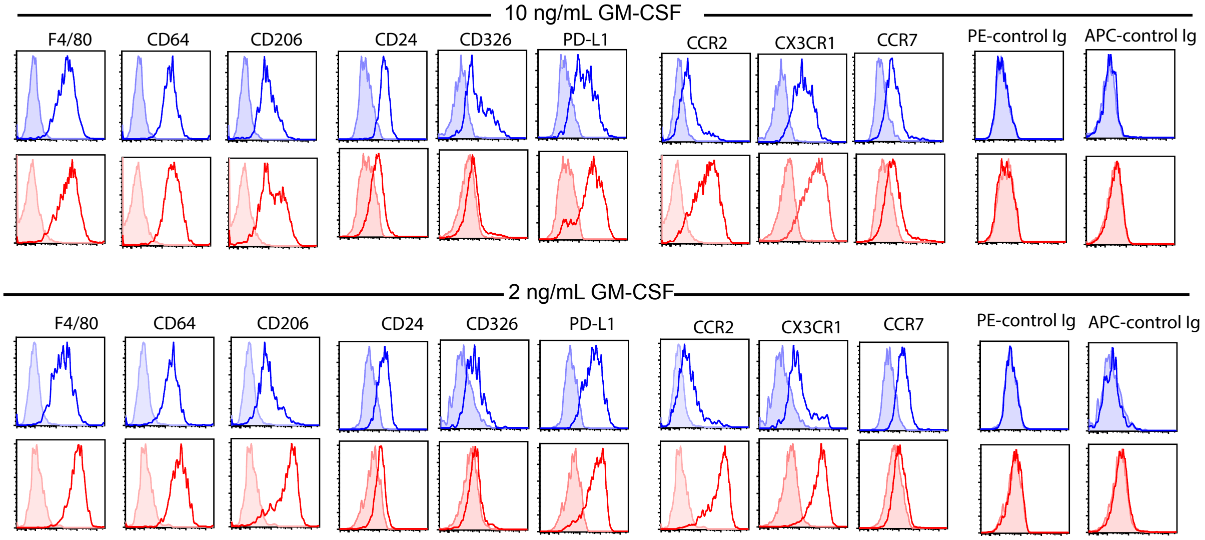

B

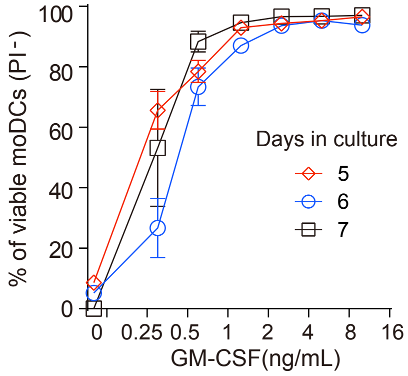

Supplementary Figure 2:

A. GM-CSF doses impact on DC generation in vitro. BM cells were cultured with high (10 ng/mL) and low dose (2 ng/mL) of GM-CSF for 7 days. Cultures were harvested and analyzed for cell composition. Gated moDCs ($CD11b^{lo}MHC^{hi}$) and mo/mϕ ($CD11b^{hi}MHC^{lo}$) were shown for expression of various myeloid markers. Shade area represents FMO controls.

B. The survival of moDCs is not affected by the doses of GM-CSF. Bone marrow cells were cultured in the presence of increasing concentration of GM-CSF indicated. The viability of moDCs in the cultures were examined by FACS from day 5 to day 7, and plotted as histogram.
